# Nonrandom Distribution of miRNAs Genes and Single Nucleotide Variants in Keratoconus Loci

**DOI:** 10.1371/journal.pone.0132143

**Published:** 2015-07-15

**Authors:** Dorota M. Nowak, Marzena Gajecka

**Affiliations:** 1 Department of Genetics and Pharmaceutical Microbiology, Poznan University of Medical Sciences, Poznan, Poland; 2 Institute of Human Genetics, Polish Academy of Sciences, Poznan, Poland; University of Florida, UNITED STATES

## Abstract

Despite numerous studies, the causes of both development and progression of keratoconus remain elusive. Previous studies of this disorder focused mainly on one or two genetic factors only. However, in the analysis of such complex diseases all potential factors should be taken into consideration. The purpose of this study was a comprehensive analysis of known keratoconus loci to uncover genetic factors involved in this disease causation in the general population, which could be omitted in the original studies. In this investigation genomic data available in various databases and experimental own data were assessed. The lists of single nucleotide variants and miRNA genes localized in reported keratoconus loci were obtained from Ensembl and miRBase, respectively. The potential impact of nonsynonymous amino acid substitutions on protein structure and function was assessed with PolyPhen-2 and SIFT. For selected protein genes the ranking was made to choose those most promising for keratoconus development. Ranking results were based on topological features in the protein-protein interaction network. High specificity for the populations in which the causative sequence variants have been identified was found. In addition, the possibility of links between previously analyzed keratoconus loci was confirmed including miRNA-gene interactions. Identified number of genes associated with oxidative stress and inflammatory agents corroborated the hypothesis of their effect on the disease etiology. Distribution of the numerous sequences variants within both exons and mature miRNA which forces you to search for a broader look at the determinants of keratoconus. Our findings highlight the complexity of the keratoconus genetics.

## Introduction

Keratoconus (KTCN, OMIM 148300) is an eye disorder in which the cornea becomes cone-shaped because of weakening and thinning of its central part, which results in altered refractive powers, and loss of visual acuity. Its prevalence has been estimated to be 1:2.000 in the general population.[1] Most diagnosed cases are sporadic, but also familial form of KTCN is observed.[2] Despite numerous studies, the causes of both development and progression of this disease remain elusive. KTCN is thought to be a multifactorial disorder, which involves the participation of both environmental and genetic factors. Among the environmental factors, frequent eye rubbing[3] and contact lenses wearing[4] as well UV[5,6] are mentioned. Also, in some reports, coexistence of KTCN with atopy and allergy was presented.[7–10]

Linkage studies of families originated from various populations have led to the identification of several loci linked to KTCN on many different chromosomes.[2] However, only few reports indicated causative genes in these loci. Such example is the gene encoding miR-184, located within the 15q22-q25 locus.[11] Another example is *DOCK9* gene, located on chromosome 13q32, in which a mutation segregated with KTCN phenotype was identified.[12]

The analysis of singular candidate genes is another approach used in keratoconus studies. One of the main candidate genes for keratoconus was *VSX1*, which protein product plays a role in craniofacial and ocular development. Numerous mutations of *VSX1* gene (e.g. R166W, L159M, D144R and H244R) have been identified in patients with KTCN phenotype.[13–17] However, these mutations have been not confirmed in other populations as responsible for KTCN causation.[18,19] *SOD1* gene has been studied as strong KTCN candidate gene also. *SOD1* encodes a major cytoplasmic antioxidant enzyme that metabolizes superoxide radicals and provides a defense against oxygen toxicity. The increased levels of oxidative stress markers and the decreased antioxidant capacity and antioxidant defenses in KTCN corneas might be involved in the development of this disease.[5] A genomic deletion within intron 2 close to the 5’ splice junction of the *SOD1* gene was identified in three patients with KTCN.[20] However, studies conducted in other populations, including Slovenian,[21] Iranian[22] and Ecuadorian,[23] have not confirmed the correlation of *SOD1* sequence variants with keratoconus phenotype. In addition, study of patients from Saudi Arabia also excluded involvement of 7 bp deletion within intron 2 of *SOD1* gene in KTCN development.[24]

In genetic research of complex diseases, including keratoconus, an association study is a useful tool to identify single nucleotide polymorphisms. In this type of analysis the differences between allele frequency in cases and controls groups allow to identify a risk or protective nature of the genetic markers.[25] Recent association studies revealed a few candidate genes including *IL1B*,[26,27] *COL5A1*,[28] *HGF*,[29,30] *RAB3GAP1*,[31] and *LOX[32]* which were associated with increased KTCN risk. Although numerous studies have been performed, the available information of genetic aspects participating in KTCN causation is very limited. Keratoconus studies described above have brought a lot of information. However, these studies focused mainly on one or two genetic factors only, whereas in the analysis of such complex diseases as KTCN all potential factors should be taken into consideration, not just one or two. Because of that, the purpose of this study was a comprehensive analysis of known KTCN loci and the loci' content in order to find genetic factors involved in KTCN causation in the general population, which could be omitted in the original studies.

## Methods

### SNVs database

The list of Single Nucleotide Variations (SNVs), which are located in known KTCN loci were obtained on the basis of Ensembl Release 77.[33] For analyzed SNVs the allele frequencies were obtained from 1,000 Genomes Project.[34] In this project variation data were obtained from genomes of 1,092 individuals from 14 populations. Sequences of these genomes were constructed using a combination of low-coverage whole-genome and exome sequencing. All populations were grouped by the predominant component of ancestry: Europe (CEU, TSI, GBR, FIN and IBS), Africa (YRI, LWK and ASW), East Asia (CHB, JPT and CHS) and the Americas (MXL, CLM and PUR). The set in our analyzes, representing variations identified in the 1000 Genomes whole Phase 1, was named '1000 Genomes–All'.

### miRNA database

The list of miRNA genes, which are located in KTCN loci was obtained on the basis of miRBase Database Release 21.[35,36] This database is dedicated to the miRNA sequences, their annotations, and the description, including information on identification methods and location in the genome. The used version contains sequences of 24,521 miRNA's precursors, which are expressed to 30,424 mature miRNAs.

### Analysis of miRNA target prediction

A search of an online database miRDB[37] containing predicted miRNA binding sites in animals was carried out. Stored in the file miRDB predicting genes which affect the expression of specific miRNAs have been identified using an algorithm MirTarget2. This algorithm has been developed through the analysis of thousands of genes regulated by miRNAs using support vector machines (SVM). All probable binding sites were assigned a value score of between 50 and 100. The higher the score is the more reliable prediction of binding sites for each miRNA was estimated. The authors of miRDB recommended the score ≥ 80 to consider it as most likely to indicate real target gene.[37] With score value lower than 60 it is recommended to confirm the binding site with the literature data. For further analyzes we selected binding sites with score value of 80 or greater.

### Candidate gene prioritization

For selected protein genes the ranking list was performed. For this purpose ToppGene Suite was used, which results are based on topological features in protein-protein interaction network.[38] This tool is a one-stop portal for gene list functional enrichment, candidate gene prioritization using either functional annotations or network analysis, and both identification and prioritization of novel disease candidate genes in the interactome. Extended versions of the PageRank and HITS algorithms, and the K-Step Markov method were applied to prioritize disease candidate genes in a training-test schema. ToppGene Suite uses a list of known disease-related genes from earlier studies as a training set, and the rest of the known genes as a test list, then performs large-scale cross validation to rank the candidate genes and also to evaluate and compare the performance of the approach. In this study, as a training set the following genes were used: *DOCK9*, *IPO5*, *STK24*,[12] *VSX1*,[15] *SOD1*,[20] *TGFBI*,[39] *HGF*, [29] *RAB3GAP1*,[31] *LOX*,[32] *FOXO1*,[40] *ZNF469*,[41] *MIR184*,[11] *ZEB1*, *COL5A1*,[28] *IL1RN*, and *SLC4A11.[42]*

### Protein-protein interactions network analysis

For proteins encoded by genes from ToppGene Suite training set and genes *SULF1*, *CCDC80*, *FARP1*, *PDGFRB*, and *VCAN*, protein-protein interactions were evaluated with usage of the STRING 9.1,[43] which permitting for identification of the known protein-protein interactions, as well as the predicted ones. The STRING 9.1 tool was used to analyze the data available in the databases containing information about the physical interactions and biological pathways: MINT, HPRD, BIND, DIP, BioGRID, KEGG, Reactome, IntAct, EcoCyc, NCI-Nature Pathway Interaction Database, and GO. Whereas, SGD database, OMIM, FlyBase, and PubMed were used to identify frequent co-occurrence of protein's names and their genes in scientific publications.

### Amino acid substitutions analysis

The potential impact of nonsynonymous amino acid substitutions on protein structure and function was assessed with PolyPhen-2 v.2.1.0 and SIFT v.4.0.3b. The PolyPhen-2 algorithm predicts which missense changes affect structure and function of the protein. PolyPhen-2 uses Position-Specific Independent Counts (PSIC) software to assign profile scores. These scores are the likelihood of a given amino acid occurring at a specific position, compared with likelihood of this amino acid occurring at any position (background frequency). The SIFT analytic tool evaluates conserved positions and calculates a score for the amino acid change at a particular position. A score greater than 0.05 was considered as tolerated for the protein structure.

### Prediction of SNVs' effect on RNA secondary structure

The effect of SNVs on RNA secondary structure was predicted using RNAsnp (version 1.1).[44] The wild-type mRNA sequences and the SNVs were given as input along with default parameters of RNAsnp. For each SNV, RNAsnp considered a window of +/−100 nucleotides around the SNV position to 1. generate the wild-type (WT) and mutant (MT) subsequences, and 2. compute their respective base pair probability matrices. Then, the difference between the base pair probability of wild-type and mutant structure was measured using Euclidean distance (d) and was computed for local intervals with minimum length equal 50.

## Results

### Comparison of sequence variants in KTCN linkage loci to indicate causative sequence changes

Fifteen loci previously linked to KTCN (Table 1) were analyzed. The boundaries of the assessed chromosomal regions were determined on the basis of border markers from the original papers reporting the particular regions.

**Table 1:** List of analyzed KTCN loci.

No.	Locus	No. of sequence variants	No. of miRNA genes	References
1	1p36.23–36.21	90 463	7	Burdon KP, 2008
2	2p24	27 475	1	Hutchings H, 2005
3	2q13-q14.3	269 944	20	Nowak DM, 2013
4	3p14–q13	680 959	17	Brancati F, 2004
5	5q14.1-q21.3	363 466	8	Tang YG, 2005
6	5q21.2	24 708	0	Bisceglia L, 2009
7	5q32-q33	215 027	9	Bisceglia L, 2009
8	8q13.1-q21.11	186 267	4	Burdon KP, 2008
9	13q32	75 741	4	Gajecka M, 2009
10	14q24.3	36 720	3	Liskova P, 2010
11	14q11.2	69 526	3	Bisceglia L, 2009
12	15q22.33–24.2	140 100	11	Hughes AE, 2003
13	16q22.3-q23.1	93 446	1	Bisceglia L, 2009
14	20p13-p12.2	126 729	4	Nowak DM, 2013
15	20q12	20 313	1	Fullerton J, 2002

Table 1 presents number of sequences variants obtained from 1,000 Genomes for all analyzed loci. For further analysis total of 2,404,106 variants in the KTCN loci was used. This set of variants included mostly SNVs but also deletions, insertions and other sequence alternations (S1 Table). From all these variants, 20,883 were located within exons of different protein-coding genes (S2 Table). 12,217 recognized sequence changes resulted in amino acid substitutions. From those SNVs, PolyPhen-2 predicted 2,822 as probably damaging the structure of protein, and 2,260 possibly damaging, while SIFT predicted 4,046 sequences variants as deleterious for the coded proteins. In summary, 3,026 variants were indicated as damaging by both programs. Subsequently, prioritization of 289 genes carrying these variants, based on topological features, was performed. The five highest positions in the ranking had got: *SULF1* (8q13.1-q21.11), *CCDC80* (3p14-q13), *FARP1* (13q32), *PDGFRB* (5q32-q33), and *VCAN* (5q14.1-q21.3) (Table 2). Interactions between these genes and training set were analyzed by String 9.1 (S1 Fig). Only single interactions between these chosen five genes were observed. What is more, a small number of interactions in the training set, between genes from earlier KTCN studies, was also pointed. The analysis revealed that the most of the observed interactions for genes from training set were the result of the coexistence in publications devoted to keratoconus and other eye diseases. The same analysis without textmining showed much less connections between analyzed proteins (S2 Fig).

**Table 2:** Chosen SIFT analysis results. List of SNVs localized within five highest ranked genes based on prioritization analysis with ToppGene, which were predicted as probably or possibly damaging or deleterious during PolyPhen and SIFT analyzes.

Chromosome name	Position on chromosome (bp)	Variation Name	Gene	Protein allele	PolyPhen prediction	SIFT prediction
3	112 324 305	rs114697626	*CCDC80*	Y/H	probably damaging	deleterious
3	112 328 836	rs56683778	*CCDC80*	A/V	probably damaging	deleterious
5	82 786 006	rs140063016	*VCAN*	P/S	possibly damaging	deleterious
5	82 789 493	rs141008923	*VCAN*	G/R	probably damaging	deleterious
5	82 834 362	rs201704138	*VCAN*	T/N	possibly damaging	deleterious
5	82 836 706	rs201327923	*VCAN*	N/K	possibly damaging	deleterious
5	149 514 363	rs2229560	*PDGFRB*	I/T	possibly damaging	deleterious
8	70 515 476	rs118056333	*SULF1*	I/V	possibly damaging	deleterious
8	70 550 806	rs150467604	*SULF1*	T/M	probably damaging	deleterious
13	99 083 321	rs61730892	*FARP1*	H/Y	probably damaging	deleterious
13	99 092 424	rs200890628	*FARP1*	I/T	probably damaging	deleterious
13	99 092 998	rs61749894	*FARP1*	R/C	probably damaging	deleterious
13	99 100 506	rs143527821	*FARP1*	R/C	probably damaging	deleterious

### SNP analysis in Ecuadorian families, 1000 Genomes–All and AMR populations to verify familial specificity of identified variants

For linkage loci from our previous studies, allele frequencies between Ecuadorian families and 1,000 Genome Project were compared. Allele frequencies from all 1,000 Genomes populations (ALL) and Ad Mixed American (AMR) were used. Analyzed SNVs were reported in original publications, which described loci 13q32[12] and 13q34[45] in family KTCN-014, and loci 2q13-q14.3 and 20p13-p12.2 in family KTCN-019.[42] S3 Fig shows differences between populations at four KTCN loci: 13q32, 13q34, 2q13-q14.3, and 20p13-p12.2. Allele’s frequencies in Ecuadorian families and 1,000 Genomes population were comparable in majority of observed SNVs at these loci. The main differences between analyzed populations were noticed within *SLC4A11* gene at locus 20p13-p12.2. However, an allele rs2281575, which occurred with KTCN phenotype more frequently in KTCN-019 family, had similar frequencies in Ecuadorian family and 1000 Genomes populations. Therefore, observed differences in alleles frequencies of other SNVs in *SLC4A11* gene, could constitute characteristic pattern for this family only. On the contrary, in locus 13q32 the most important differences in allele frequencies between Ecuadorian and 1000 Genomes–All populations were observed for rs7995432 and rs145089138 segregated with KTCN phenotype in family KTCN-014.

### Comparison of SNP frequencies between the 1000 Genomes–All population and chosen populations from association studies

**Fig 1:**
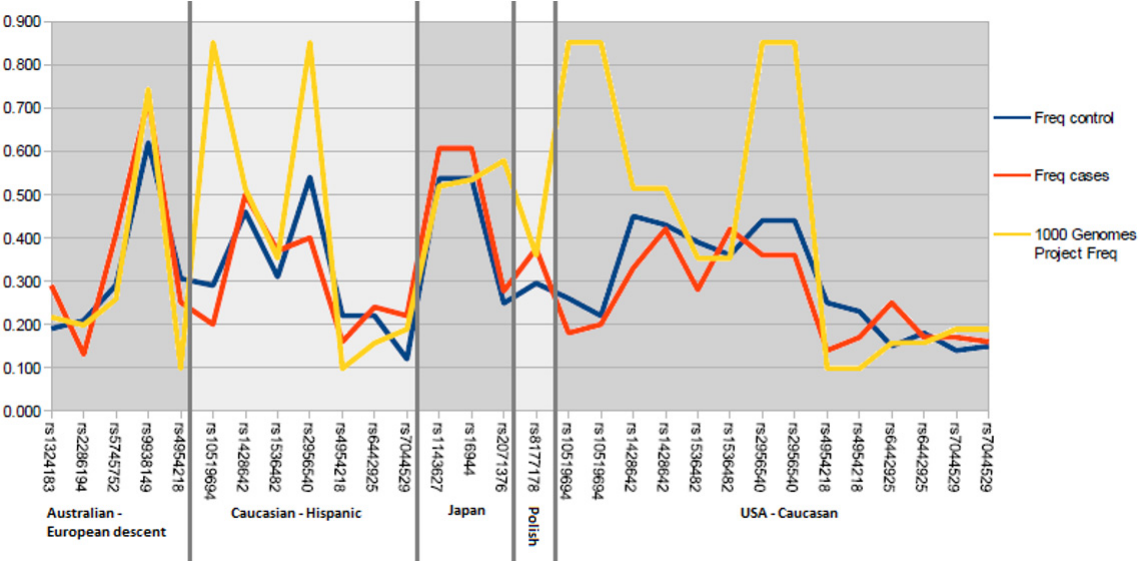
Differences in allele frequencies between analyzed populations from association studies. Vertical axis represents alleles frequencies and horizontal axix–SNVs. All SNP for each population are grouped together.

Fifteen SNVs from 8 different association studies [27,28,30–32,46,47] were analyzed (Table 3). Selected SNVs were described in original publications as significant or suggestive variants in KTCN etiology. In addition, seven sequences variants of genes associated with thinness of central cornea (TCC) were examined. Most of the populations from original reports were described as of European/Caucasian origin (Table 3). For majority of observed sequences variants, the most imortant differences in allele frequencies were identified between 1) the cases and 1,000 Genome–All population and 2) the controls and 1,000 Genome–All comparing to cases and controls within the same population. Such example is rs2071376, where obtained frequencies were 0.578 for 1000 Genomes–All, and 0.249 and 0.277 for cases and controls from Japan population (Fig 1). Low variation in frequencies in KTCN populations between cases and controls could indicate strong population specificity of the analyzed SNVs.

**Table 3:** Chosen SNVs from selected KTCN association studies involving experiments indicating especially CCT genes.

Gene	rs number	Allel	1000 Genomes ALL Freq	Freq control	Freq cases	Population	References
BANPZNF469*	rs9938149	A	0.743	0.620	0.720	Australian—European descent	Sahebjada et al., 2013
COL5A1*	rs1536482	A	0.353	0.310	0.370	Caucasian, Hispanic	Li et al., 2013
*COL5A1*	rs1536482	A	0.353	0.390	0.280	USA–Caucasian	Li et al., 2013
*COL5A1*	rs1536482	A	0.353	0.360	0.420	USA–Caucasian	Li et al., 2013
*COL5A1*	rs7044529	T	0.189	0.120	0.220	Caucasian, Hispanic	Li et al., 2013
*COL5A1*	rs7044529	T	0.189	0.140	0.170	USA–Caucasian	Li et al., 2013
*COL5A1*	rs7044529	T	0.189	0.150	0.160	USA–Caucasian	Li et al., 2013
*HGF*	rs2286194	A	0.198	0.209	0.131	Australian—European descent	Sahebjada et al., 2014
*HGF*	rs5745752	A	0.258	0.291	0.409	Australian—European descent	Sahebjada et al., 2014
*IL1A*	rs2071376	A	0.578	0.249	0.277	Japan	Mikami et al., 2013
*IL1B*	rs1143627	T	0.519	0.537	0.607	Japan	Mikami et al., 2013
*IL1B*	rs16944	C	0.534	0.537	0.607	Japan	Mikami et al., 2013
*LOX*	rs10519694	T	0.149	0.290	0.200	Caucasian, Hispanic	Bykhovskaya et al., 2012
*LOX*	rs10519694	T	0.149	0.260	0.180	USA–Caucasian	Bykhovskaya et al., 2012
*LOX*	rs10519694	T	0.149	0.220	0.200	USA–Caucasian	Bykhovskaya et al., 2012
*LOX*	rs2956540	G	0.648	0.540	0.400	Caucasian, Hispanic	Bykhovskaya et al., 2012
*LOX*	rs2956540	G	0.648	0.440	0.360	USA–Caucasian	Bykhovskaya et al., 2012
*LOX*	rs2956540	G	0.648	0.440	0.360	USA–Caucasian	Bykhovskaya et al., 2012
MPDZ-NF1B*	rs1324183	A	0.217	0.190	0.290	Australian—European descent	Sahebjada et al., 2013
*RAB3GAP1*	rs1428642	A	0.514	0.460	0.500	Caucasian, Hispanic	Li et al., 2012
*RAB3GAP1*	rs1428642	A	0.514	0.450	0.330	USA–Caucasian	Li et al., 2012
*RAB3GAP1*	rs1428642	A	0.514	0.430	0.420	USA–Caucasian	Li et al., 2012
*RAB3GAP1*	rs4954218	G	0.098	0.306	0.251	Australian Caucasian	Bae et al., 2013
*RAB3GAP1*	rs4954218	G	0.098	0.220	0.160	Caucasian, Hispanic	Li et al., 2012
*RAB3GAP1*	rs4954218	G	0.098	0.250	0.140	USA–Caucasian	Li et al., 2012
*RAB3GAP1*	rs4954218	G	0.098	0.230	0.170	USA–Caucasian	Li et al., 2012
*RAB3GAP1*	rs6442925	T	0.157	0.220	0.240	Caucasian, Hispanic	Li et al., 2012
*RAB3GAP1*	rs6442925	T	0.157	0.150	0.250	USA–Caucasian	Li et al., 2012
*RAB3GAP1*	rs6442925	T	0.157	0.180	0.170	USA–Caucasian	Li et al., 2012
*TF*	rs8177178	A	0.360	0.295	0.375	Polish	Wojcik et al., 2013

* Genes associated with KTCN and corneal thickness

### Analysis of miRNA genes to identify SNPs in mature sequences and their potential influence on miRNA activity

Within the studied loci 88 genes encoding miRNAs were identified. Eighteen of miRNAs genes were located in the locus 3p14-q13 (Table 4), whereas at 5q21.2 the miRNAs genes were not observed. From the 88 analyzed miRNAs genes, 33 genes encoded two mature miRNA derived from opposite arms of the hairpin structure of pre-miRNA.

**Table 4:** SNVs in mature miRNAs.

Locus	SNV	miRNA genes	Localization in seed region
13q32	rs147240207	hsa-mir-3170	no
13q32	rs369781239	hsa-mir-623	no
13q32	rs373350242	hsa-mir-623	no
14q11.2	rs372515832	hsa-mir-208a	no
14q11.2	rs2754157	hsa-mir-208b	no
14q11.2	rs2273626	hsa-mir-4707	yes
14q11.2	rs368405695	hsa-mir-4707	no
15q22.33–24.2	rs2168518	hsa-mir-4513	yes
15q22.33–24.2	rs116034786	hsa-mir-4514	no
15q22.33–24.2	rs141006148	hsa-mir-549	no
16q22.3-q23.1	rs368772301	hsa-mir-4719	yes
1p36.23–36.21	rs35301225	hsa-mir-34a	no
1p36.23–36.21	rs369892834	hsa-mir-34a	yes
1p36.23–36.21	rs372809836	hsa-mir-4632	yes
2q13-q14.3	rs189786001	hsa-mir-3679	no
2q13-q14.3	rs139701217	hsa-mir-3679	no
2q13-q14.3	rs117721121	hsa-mir-4783	no
2q13-q14.3	rs113469098	hsa-mir-4782	no
3p14–q13	rs199975935	hsa-mir-1324	yes
3p14–q13	rs200933343	hsa-mir-1324	no
3p14–q13	rs202177109	hsa-mir-1324	no
3p14–q13	rs202232971	hsa-mir-1324	no
3p14–q13	rs199620349	hsa-mir-4273	no
3p14–q13	rs201246393	hsa-mir-4273	no
3p14–q13	rs76333414	hsa-mir-4273	no
3p14–q13	rs185664152	hsa-mir-4445	no
3p14–q13	rs76069948	hsa-mir-4445	no
3p14–q13	rs185555286	hsa-mir-4796	no
3p14–q13	rs59323834	hsa-mir-548ab	no
3p14–q13	rs146499813	hsa-mir-567	no
3p14–q13	rs369579186	hsa-mir-567	yes
3p14–q13	rs375214593	hsa-mir-567	yes
3p14–q13	rs149509568	hsa-mir-568	no
3p14–q13	rs28632138	hsa-mir-568	no
3p14–q13	rs193065146	hsa-mir-5688	no
5q32-q33	rs189565536	hsa-mir-1294	no
5q32-q33	rs370825499	hsa-mir-378a	no
5q32-q33	rs376185067	hsa-mir-378a	no
5q32-q33	rs3734050	hsa-mir-6499	yes

Subsequently, in the studied miRNA genes known SNVs were assessed. In total, in 88 miRNA genes 109 known SNVs from 1000 Genomes Project were located. However, within 36 miRNAs genes no SNVs have been observed. On the other hand, in the gene hsa-mir-4445, six known sequence variants were located, and approximately 1 was the average number of known SNVs in the analyzed miRNA genes. In addition, among all examined SNVs, 29 were located within the sequence of the mature miRNA (Table 4) and the remaining 92 variants were in other regions of pre-miRNA stem-loop structures.

The miRDB base was searched to determine the expected binding sites for mature miRNAs encoded by the examined genes. The criterion of score ≥ 80 was fulfilled in 4992 genes coding proteins. For 116 protein coding genes the score was equal 100, and predictions of 248 targets had score of 99. For 545 proteins the predictions the obtained score was equal 80. Majority of genes was predicted as target for only one miRNA from the list. However, *AFF4* was pointed by 14 of the analyzed miRNAs, while for 10 miRNAs as a target the *MIER3* and *DENND1B* genes have been predicted. On the other hand, on average, more than 78 probable binding sites for each miRNAs were indicated. Additionally, for hsa-miR-4719, 262 predicted binding sites fulfilled including criteria, and for hsa-miR-7846-3p, one protein interaction was listed only. However, genes *VSX1*, *SOD1*, previously strongly correlated with KTCN phenotype, were not present among the genes that fulfilled the established criteria. Nevertheless, genes located in locus 13q32, *MBNL* and *ZIC5*, have been predicted as target for miR-548ab, miR-5688 (*MBNL2)* and miR-568 (*ZIC5*).

In 36 miRNAs genes known SNVs in the region of mature miRNA are localized. For these 36 miRNAs over 400 target protein genes were indicated by miRDB. Subsequently, for these genes the ranking based on topological features in protein-protein interaction network was created. From all tested genes, the highest three ranks had: *SMAD2*, *CAND1*, and *VHL*.

### Prediction of SNVs' effect on RNA secondary structure

**Fig 2:**
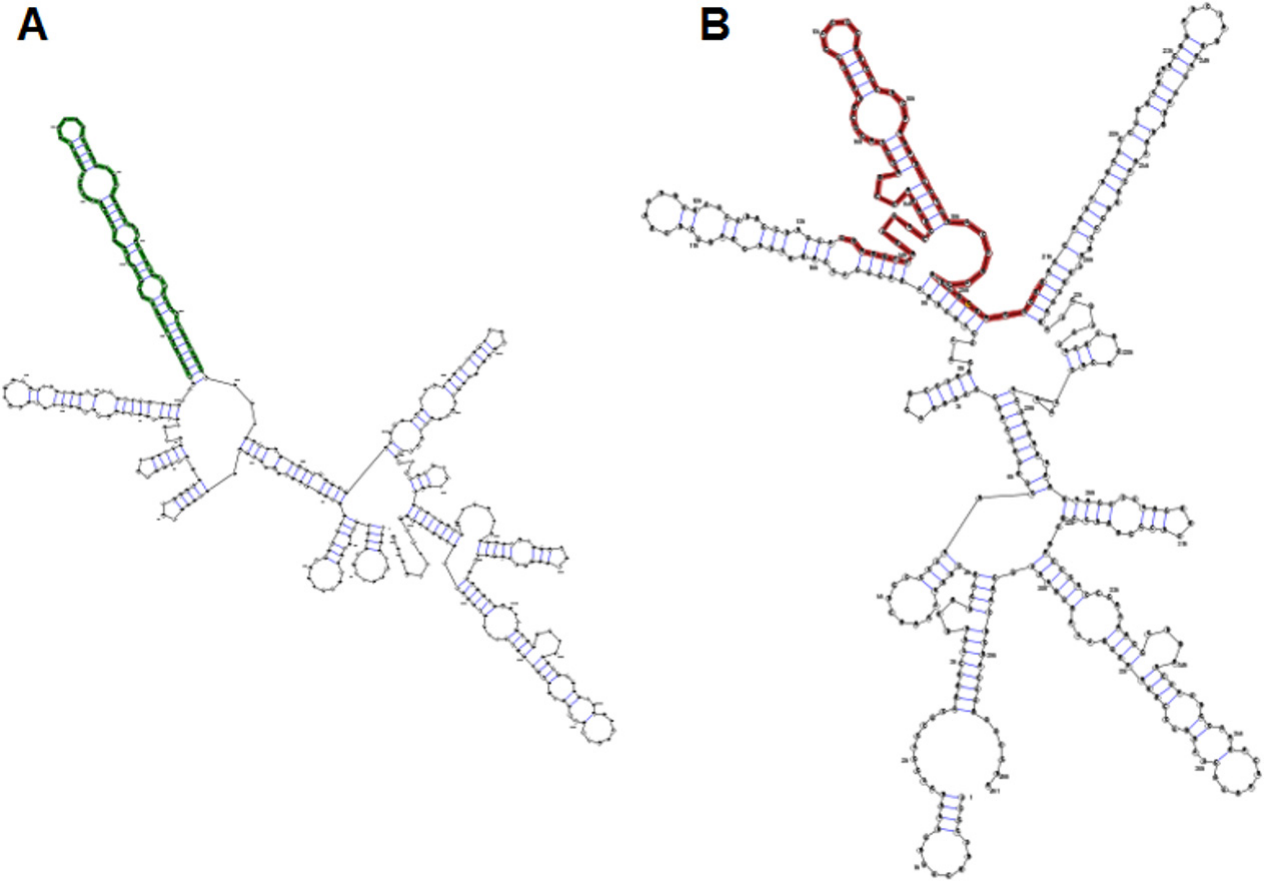
Visualizing the secondary structure in planar graph representation. In bold RNA sequences analyzed by RNAsnp. A–Wild-type sequence, B—sequence with rs202177109

For 79 analyzed SNVs within miRNA genes the prediction of RNA secondary structure was evaluated. Only 20 SNVs were reported as causing significant changes in RNA structures. Seven of 20 known SNVs in hsa-mir-1324 gene were predicted to cause changes in secondary structure (Fig 2).

## Discussion

KTCN is considered as a multifactorial disorder, in which both environmental and genetic factors are involved. The identification of numerous KTCN loci in different populations indicates polygenic bases of the disease. However, only for a few loci candidate genes were identified. Interesting example was the finding of mutation in the seed region of miR-184, located at the 15q22-q25 locus.[11]

In this study numerous sequences variants localized in known KTCN loci were analyzed. Analysis of 13 loci revealed 1,045,902 SNVs in 1,000 Genome database located within over two thousands of various genes. We further analyzed genes, in which sequence variants cause amino acid substitution to point at SNVs potentially causative in KTCN etiology. From more than one million variants, 12,217 cause amino acid substitution. In addition, 3,026 SNVs were indicated by both SIFT and PolyPhen-2 as deleterious. Subsequently, for 289 genes, in which these SNVs were located, the ranking based on topological features in protein-protein interaction network was created. From all tested genes, *SULF1*, *CCDC80*, *FARP1*, *PDGFRB*, and *VCAN* have got the highest five ranks. These genes include the ones previously assessed in KTCN studies and those recognized in this investigation.

*FARP1* gene has been already evaluated in a study of locus 13q32 in a large multigenerational Ecuadorian family.[23] What is more, the highest linkage peak was identified exactly in this gene, as described in the original paper.[23] However, none of sequences variants observed in *FARP1* was segregating with KTCN phenotype in that study.[23] In addition, SNPs presented in Table 2 were not observed in original study in Ecuadorian population. Protein encoded by *FARP1* gene regulates dendritic filopodial dynamics in immature neurons, indicating a role in synapse formation. Interesting is fact that in human *RNF113B* gene was retroposed in opposite direction into a first intron of *FARP1* gene.[46] What is more, a gene coded miR-3170 is located within intron 1 of *FARP1*.[47] Example of *FARP1* has shown that a simply analysis of coding regions was not sufficient.

As the aspect of corneal wound healing is frequently discussed in KTCN etiology the appearance of two genes possibly involved in corneal wound healing in the ranking, *SULF1* and *PDGFRB* is not surprising. Earlier studied showed that *SULF1* underwent a 40-fold upregulation in blood vessels associated with wounded skin in human[48] and the protein product of *SULF1* gene was involved in promotion of the migration of corneal epithelial cells during wound healing.[49] Similarly, studying *PDGFRB* gene, Hoppenreij et al. suggested that the presence of PDGFR in human corneal epithelium, fibroblasts, and endothelium and the mitogenic effects of PDGF on corneal cells indicated that PDGF might play a role in corneal wound healing.[50] *PDGFRB* gene encodes a cell surface tyrosine kinase receptor for members of the platelet-derived growth factor family.

To date role of *CCDC80* and *VCAN* genes has not been indicated in KTCN etiology. *CCDC80* encodes a protein involved in the induction of C/EBPα and peroxisome proliferator-activated receptor γ (PPARγ),[51] which acts as a negative regulator in immune cells. In addition, PPARγ agonist markedly suppresses both expression of thymic stromal lymphopoietin in the skin and maturation and migration of dendritic cells in a mouse model of atopic dermatitis.[52] *CCDC80* gene was also listed among eight loci associated with atopic dermatitis in the Japanese population.[53] *VCAN* gene encodes a versican, which is an extracellular matrix protein and a component of the vitreous, where it is likely to be involved in maintenance and structural integrity.[54] The mutation of the *VCAN* gene is a cause of Wagner syndrome, which manifests retinal detachment, myopia, presenile cataract, night blindness with progressive chorioretinal atrophy, extensive retinal pigment clumping, ectopic foveae, inverted papilla, uveitis, and glaucoma.[55]

In this study some aspects were examined based on our experimental data obtained from previous studies in Ecuadorian families. Allele frequencies in Ecuadorian population evaluated in our previous studies and frequencies from ALL and AMR populations were compared. The aim of this analysis was to identify the cause of allele’s frequencies disparity between populations. More frequent occurrence of one allele might be caused by family-based specificity, population specificity, as well as observed KTCN phenotype. In locus 13q32 the highest differences between Ecuadorian family and 1000 Genomes population, were present for SNVs (rs7995432 in *DOCK9* and rs145089138 in *IPO5*) segregated with KTCN phenotype in family KTCN-014. However, the rest of analyzed SNVs in 13q32 had comparable frequencies in both Ecuadorian family and 1000 Genomes population. That suggests strong involvement of these sequence variants in KTCN etiology. On contrary, overall allele frequencies of rs2281575 within *SLC4A11* gene from locus 20p13-p12.2, which occurred with KTCN phenotype more frequently were comparable with data from 1000 Genomes populations. This could indicate both small effect of this SNV on KTCN development and family-based specificity, what makes this result difficult to replicate in other population or even other family. These results confirm the requirement for a careful analysis of allele frequencies in different populations and the need to compile them with frequencies in general population.

Previous studies on the genetics of keratoconus have been mainly based on protein-coding sequences, and obtained results indicated that this approach was insufficient. Therefore, we analyzed miRNAs genes localized within know KTCN loci. Sequence variants in miRNAs are difficult to analyze, because the changes do not produce obvious functional alterations in the way that mutations in protein-coding genes do. In total, we analyzed 121 miRNAs encoded by 88 genes. Our results demonstrated the presence of numerous miRNA genes in KTCN loci. What is more, many SNVs in region coding mature miRNA have been recognized. Such sequence variants may in significant manner affect the function of individual miRNA. Subsequently, these miRNAs seem more interesting for further investigation. Analysis of these SNVs in KTCN patients could reveal new elements of KTCN etiology. Subsequently the influence on pre-miRNA structure and efficiency of miRNA binding should be studied. In addition, the analysis of predicted targets for these miRNAs has revealed genes already discussed in previous KTCN studies. Such examples are genes located at 13q32, *MBN*L and *ZIC5,[12]* predicted as target for miR-548ab (*MBNL*2), miR-5688 *(MBNL*2) and miR-568 *(ZIC*5). However, the coding sequence variants identified in *ZIC*5 and *MBNL*2 have been observed in both affected and unaffected individuals in the discovery family.[12] In consequence, these genes were not further discussed as causative in KTCN.[12] However, in the original study only exons, exon-intron boundaries, and UTRs were analyzed. Wide spectrum of splicing and sequences involved in regulation of expression has not been studied in this genes.[12] The miR-548ab and miR-5688 are located in locus 3p14–q13, which was identified in Italian KTCN family.[56] Brancati et al. in their study analyzed *COL8A*1 as a candidate gene, but they have not detected any pathogenic mutations in the coding sequences. Furthermore, in locus 3p14–q13 17 miRNAs genes are localized, however to this date, none of them was analyzed in context of KTCN development. Also, miR-568 is mapped in locus 8q13.1-q21.11, which was identified in the extended KTCN Caucasian family of Western European descent.[57] However, the locus on chromosome 8 and locus 1p36.23–36.21 were recognized together pointed by digenic linkage analysis and they reached significant LOD in simultaneous analysis only. However, because none of loci was statistically significant in independent analyzes, further studies of those two loci including genes interactions and miRNAs genes should be performed.

For further analysis, for genes predicted as targets for miRNAs from KTCN loci were ranked based on topological features in protein-protein interaction network. The three highest ranked genes were *SMAD2*, *CAND1*, and *VHL*. *SMAD2* gene was predicted as target for miR-568. SMAD proteins are signal transducers and transcriptional modulators that mediate multiple signaling pathways. SMAD2 protein mediates the signal of the transforming growth factor beta (TGF-β), and thus regulates multiple cellular processes, such as cell proliferation, apoptosis, and differentiation. The analysis of cornea from autopsy control and keratoconus patients using RT-PCR exhibited elevated messenger ribonucleic acid levels of Smad2 and TGF-β in severe keratoconus corneal epithelium.[58] The participation of the TGF-β pathway in the modulation and production of extracellular matrix may suggest involvement in the pathogenesis of KTCN, either in a causative role or a secondary repair response leading to structural changes in this disease. Additionally, Brown et al. hypothesized that Dock9 could play a role in the activation of Cdc42 and affect TGF-β1-mediated Smad-dependent transcriptional responses.[59] Therefore we come back again to a *DOCK9* gene in which mutation in KTCN was postulated as causative.[12]

*SMAD2* gene encodes protein that is a signal transducer and transcriptional modulator that mediate multiple signaling pathways. Phosphorylated Smad2 (pSmad2) forms a complex with the mediator Smad4 and is translocated into the nucleus, where acts as a transcription factor for multiple TGFβ-dependent genes. In their study, Engler et al., showed an increase of tgfb2 and pSmad2 signal in the epithelium of severe keratoconus cases.[58]

Most of identified mutations in protein's genes have not been replicated in other populations. However, some of miRNAs revealed in this study may regulate expression of genes located in known KTCN loci. Such example may be miR-548ab, miR-5688, and miR-568, for which possible interactions with genes *MBNL2* and *ZIC5* from locus 13q32 have been predicted. This may suggest necessity to reanalyze known loci and sequence in full genes localized within. Nowadays, a good solution will be application of next generation sequencing (NGS) in those additional analyses.

However, expression in human cornea of numerous analyzed in this study miRNAs remains to be examined. Ryan et al. reported at least 31 miRNAs expressed in adult mouse corneas by miRNA microarray profiling.[60] Additionally, Lee et al. found differential expressions of 18 microRNAs between human limbal-peripheral and central corneal epithelia.[61] Among them, miR-143 and miR-145 were expressed predominantly in the limbal epithelium but at very low levels in the central corneal epithelium. What is more, Lee et al. hypothesized that miR-145 could be an important regulatory molecule for human corneal epithelial differentiation. The cells in their study developed to thinner and defective epithelium *in vitro*. It was suggested that morphological alteration could be caused by miR-145 via the direct targeting on *ITGB8*. Very well is also characterized expression of miR-184, which was reported in earlier research as the highest expressed miRNA in the cornea.[60] Hughes et al., hypothesized that miR-184 with the r.57c>u mutation failed to compete with miR-205 for overlapping target sites on the 3′ UTR of two target genes, *INPPL1* (inositol polyphosphate phosphatase-like 1) and *ITGB4* (integrin beta 4). These two target genes are involved in corneal healing after injury as the principal component of corneal basal epithelial hemidesmosomes.[11]

In KTCN studies there is one another aspect which has not been fully examined. Previous reports suggested that oxidative stress may be involved in KC.[62–64] The cornea is a transparent avascular tissue that absorbs approximately 80% of the incident ultraviolet B (UVB) light,[65] making it highly sensitive and vulnerable to damage from free radicals and reactive oxygen species (ROS). Oxidative stress is defined as the imbalance between the systemic manifestation of ROS and a biological system's ability to detoxify or repair any resulting damage from the reactive intermediates.[65,66] Recent study of in vitro model showed that cells derived from keratoconus patients were under oxidative stress.[67]

One of the main sources of reactive oxygen is the leakage of activated oxygen from mitochondria during the metabolic pathway of oxidative phosphorylation. However, till this date only one study analyzed full mitochondrial genome in KTCN patients. Abu-Amero et al. analyzed mitochondrial genome in 27 KTCN individuals and 100 controls (Abu-Amero et al., 2014).[68] Within numerous sequences variants, 10 of them were indicated as pathogenic and not observed in healthy controls. However, most of the observe changes were present in single individuals. The mitochondrial genome is highly mutated and one must be extremely careful in assigning pathogenic status to various sequence changes. Nevertheless, previous studies had indicated that oxidative stress, [69] mitochondrial dysfunction,[70] and mtDNA damage[71] may have a role in the development and progression of KTCN. Because of that, mitochondrial aspects should be not omitted in KTCN studies.

The obtained results showed high specificity for the populations in which causative sequence variants have been identified. In addition, confirmed the possibility of links between previously analyzed loci, e.g. MIR568 and *ZIC5*. In general, the miRNA genes themselves have been not sufficiently investigated in keratoconus. Increasing number of reports indicating involvement of genes associated with oxidative stress and inflammatory agents might confirm the hypothesis of their effect on the disease etiology.

Analysis of genetic factors in KTCN is a complex task. Beside the search for sequence variants in coding regions of candidate genes other elements creating the network of interactions should be evaluated. The comprehensive analysis of various keratoconus aspects will reduce the risk of missing important genetic factors that may affect the development of the disease.

## Supporting Information

S1 Fig Network of protein-protein interactions of proteins encoded by genes from the training set (green circles) and five highest ranked genes based on prioritization analysis with ToppGene program (dark blue circles). Protein connected lines indicate type of feature used by STRING for prediction of interactions: black–co-expression, pink–experiments, dark blue–co-occurrence, light blue–interactions described in databases, brown–textmining, and gray–homology.
(DOC)

S2 Fig Network of protein-protein interactions of proteins encoded by genes from the training set (green circles) and five highest ranked genes based on prioritization analysis with ToppGene program (dark blue circles). Protein connected lines indicate type of feature used by STRING for prediction of interactions: black–co-expression, pink–experiments, dark blue–co-occurrence, light blue–interactions described in databases, and gray–homology.
(DOC)

S3 Fig Differences in alleles frequencies between KTCN families and 1000 Genomes populations for loci: A) 13q32, B) 13q34, C) 2q13-q14.3, and D) 20p13-p12.2. (DOC)

S1 Table List of sequences variations within KTCN loci according 1000 Genomes Project. (DOC)

S2 Table Localization in genome of sequences variations within KTCN loci. (DOC)
