# Supplementary material for: Nonrandom Distribution of miRNAs Genes and Single Nucleotide Variants in Keratoconus Loci
Source: PLoS One. 2015 Jul 15;10(7):e0132143. doi: 10.1371/journal.pone.0132143 (PMC4503774; doi:10.1371/journal.pone.0132143)

**S2 Fig.** **Network of protein-protein interactions of proteins encoded by genes from the training set (green circles) and five highest ranked genes based on prioritization analysis with ToppGene program (dark blue circles).** Protein connected lines indicate type of feature used by STRING for prediction of interactions: black – co-expression, pink – experiments, dark blue – co-occurrence, light blue – interactions described in databases, and gray – homology


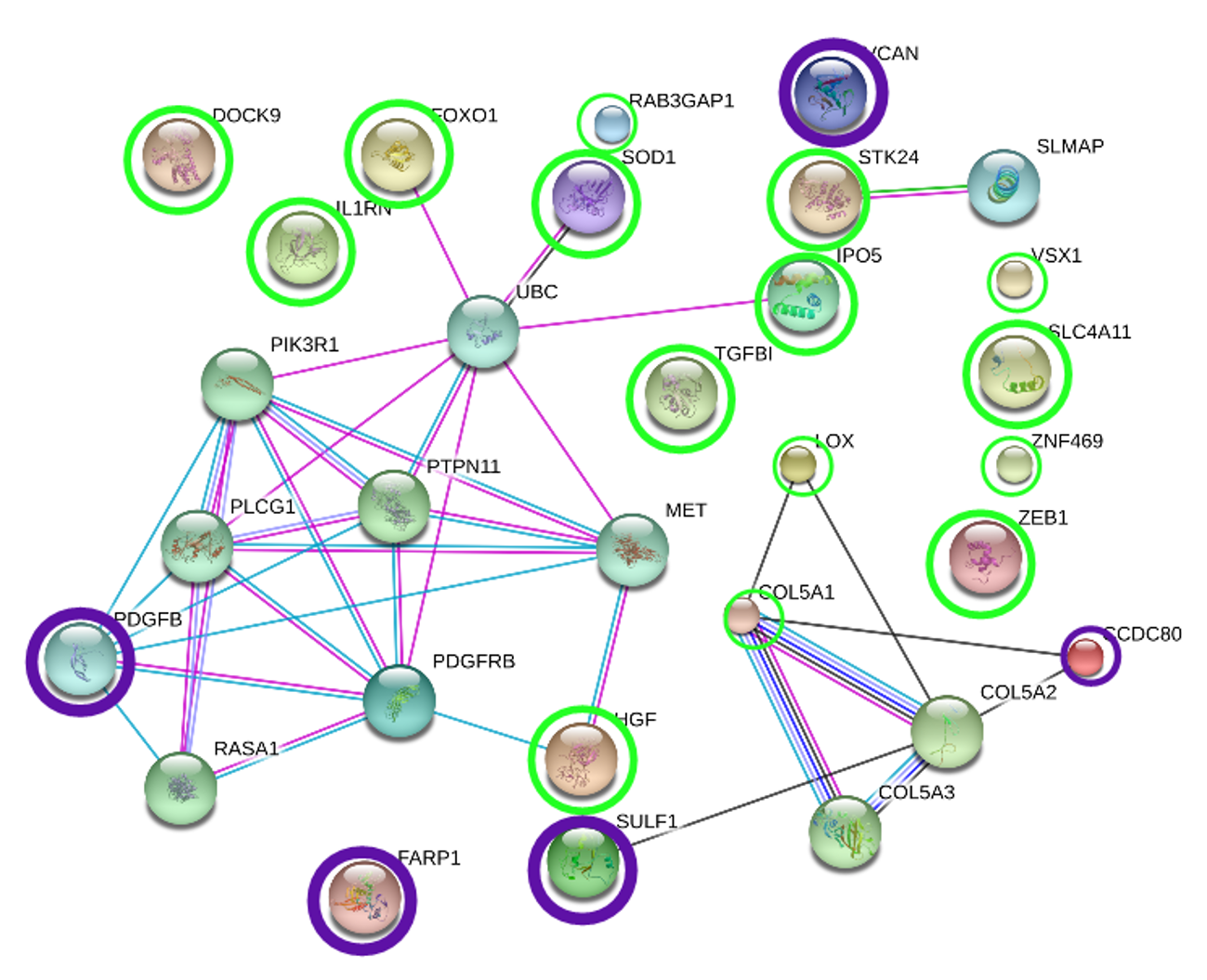

Supplement: S2 Fig — Protein connected lines indicate type of feature used by STRING for prediction of interactions: black–co-expression, pink–experiments, dark blue–co-occurrence, light blue–interactions described in databases, and gray–homology. (DOC) [file pone.0132143.s002.doc]
