# Supplementary material for: Nonrandom Distribution of miRNAs Genes and Single Nucleotide Variants in Keratoconus Loci
Source: PLoS One. 2015 Jul 15;10(7):e0132143. doi: 10.1371/journal.pone.0132143 (PMC4503774; doi:10.1371/journal.pone.0132143)

**S3 Fig. Differences in allele frequencies between KTCN families and 1000 Genomes populations for loci:** A) 13q32, B) 13q34, C) 2q13-q14.3, and D) 20p13-p12.2


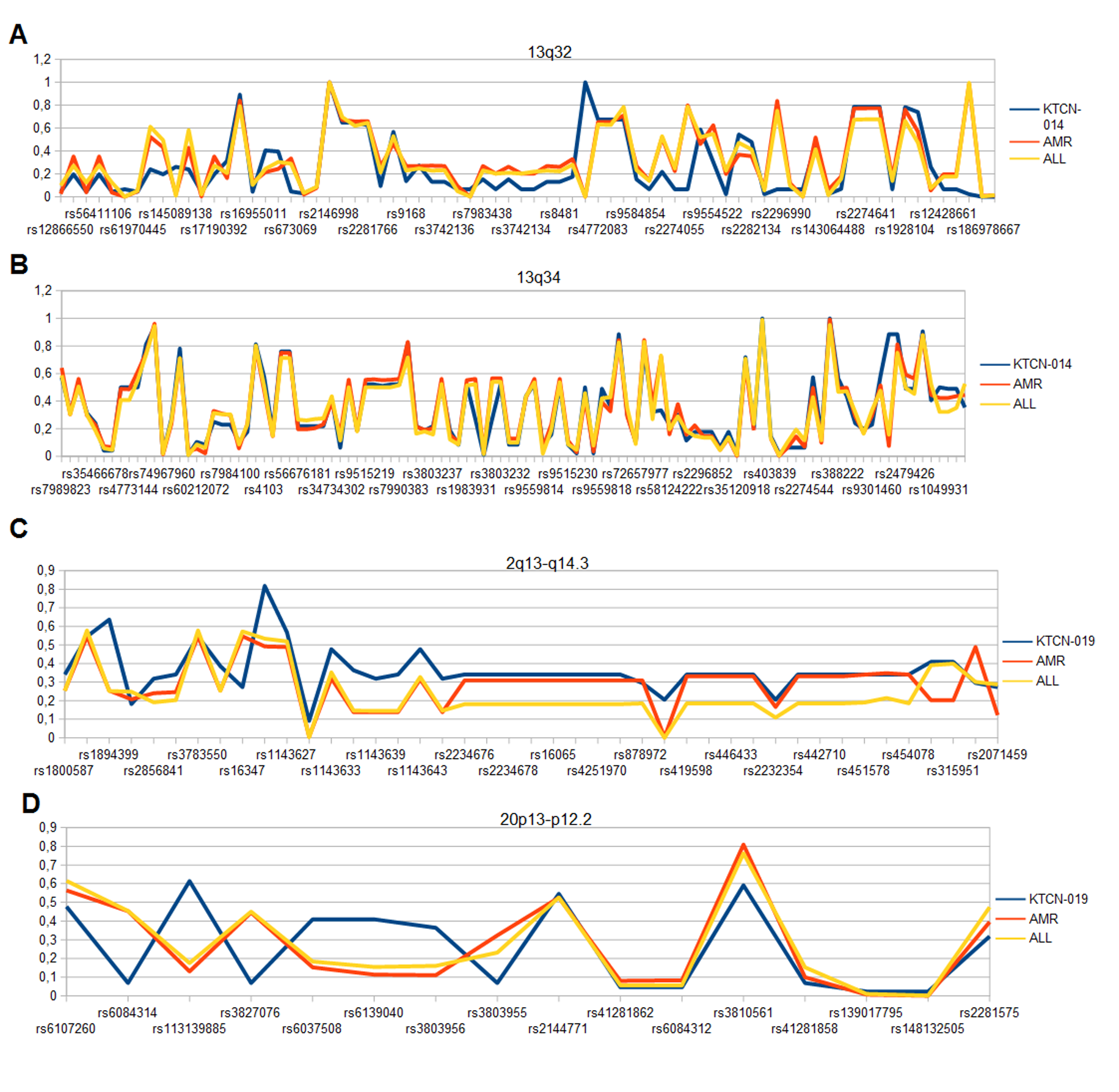

Supplement: S3 Fig — (DOC) [file pone.0132143.s003.doc]
