# Supplementary material for: Nonrandom Distribution of miRNAs Genes and Single Nucleotide Variants in Keratoconus Loci
Source: PLoS One. 2015 Jul 15;10(7):e0132143. doi: 10.1371/journal.pone.0132143 (PMC4503774; doi:10.1371/journal.pone.0132143)
Supplement: S1 Table — (DOC) [file pone.0132143.s004.doc]

**S1 Table. List of sequence variation within KTCN loci according to 1000 Genomes Project**

| **Type of sequences variations** | | **No.** |
| --- | --- | --- |
| SNVs |  | 2 343 741 |
| insertions |  | 22 905 |
| deletions |  | 36 630 |
|  | Total: | 2 403276 |

*A sequence alteration where the length of the change in the variant is the same as that of the reference.
