# Supplementary material for: Nonrandom Distribution of miRNAs Genes and Single Nucleotide Variants in Keratoconus Loci
Source: PLoS One. 2015 Jul 15;10(7):e0132143. doi: 10.1371/journal.pone.0132143 (PMC4503774; doi:10.1371/journal.pone.0132143)
Supplement: S2 Table — (DOC) [file pone.0132143.s005.doc]

**S2 Table. Localization of sequence variation in genome within KTCN loci**

| **Localization in genome** | **Chromosome** | **No.** |
| --- | --- | --- |
|  | Total | 99 516 |
|  | 1 | 5 270 |
|  | 2 | 11 718 |
|  | 3 | 19 905 |
|  | 5 | 21 635 |
| Downstream of gene | 8 | 6 222 |
|  | 13 | 2 413 |
|  | 14 | 13 529 |
|  | 15 | 6 704 |
|  | 16 | 3 427 |
|  | 20 | 8 693 |
|  | Total | 94 131 |
|  | 1 | 5 080 |
|  | 2 | 11 536 |
|  | 3 | 18 579 |
|  | 5 | 19 647 |
| Upstream gene | 8 | 5 374 |
|  | 13 | 2 554 |
|  | 14 | 13 175 |
|  | 15 | 6 561 |
|  | 16 | 3 575 |
|  | 20 | 8 050 |
|  | Total | 949 734 |
|  | 1 | 27 186 |
|  | 2 | 155 460 |
|  | 3 | 323 223 |
|  | 5 | 223 025 |
| Intergenic variants | 8 | 78 570 |
|  | 13 | 19 413 |
|  | 14 | 23 144 |
|  | 15 | 21 583 |
|  | 16 | 28 966 |
|  | 20 | 49 164 |
|  | Total | 9 531 |
|  | 1 | 303 |
|  | 2 | 1 138 |
|  | 3 | 2 174 |
|  | 5 | 2 219 |
| Non-coding transcript | 8 | 752 |
|  | 13 | 240 |
|  | 14 | 862 |
|  | 15 | 806 |
|  | 16 | 363 |
|  | 20 | 674 |
|  | Total | 8 666 |
|  | 1 | 831 |
|  | 2 | 749 |
|  | 3 | 1 140 |
|  | 5 | 1 818 |
| Protein-coding; Synonymous variants | 8 | 372 |
|  | 13 | 242 |
|  | 14 | 1 256 |
|  | 15 | 947 |
|  | 16 | 437 |
|  | 20 | 87 |
|  | Total | 12 217 |
|  | 1 | 996 |
|  | 2 | 1 139 |
|  | 3 | 1 925 |
|  | 5 | 2 644 |
| Protein-coding; Missense variants | 8 | 450 |
|  | 13 | 252 |
|  | 14 | 1 975 |
|  | 15 | 947 |
|  | 16 | 569 |
|  | 20 | 1 075 |
|  | Total | 25 454 |
|  | 1 | 1 655 |
|  | 2 | 2 575 |
|  | 3 | 3 529 |
|  | 5 | 6 095 |
| 3'UTR variants | 8 | 1 520 |
|  | 13 | 835 |
|  | 14 | 2 683 |
|  | 15 | 3 296 |
|  | 16 | 1 061 |
|  | 20 | 2 206 |
|  | Total | 5 583 |
|  | 1 | 299 |
|  | 2 | 444 |
|  | 3 | 959 |
|  | 5 | 1 141 |
| 5'UTR variants | 8 | 313 |
|  | 13 | 124 |
|  | 14 | 902 |
|  | 15 | 708 |
|  | 16 | 299 |
|  | 20 | 271 |
|  | Total | 1 198 208 |
|  | 1 | 307322 |
|  | 2 | 112 647 |
|  | 3 | 309 471 |
|  | 5 | 307 322 |
| Intron variants | 8 | 92 679 |
|  | 13 | 49 662 |
|  | 14 | 48 685 |
|  | 15 | 98 291 |
|  | 16 | 54 767 |
|  | 20 | 75 855 |
|  | Total | 82 |
|  | 1 | 7 |
|  | 2 | 5 |
|  | 3 | 14 |
|  | 5 | 13 |
| Stop gained variants | 8 | 1 |
|  | 13 | 4 |
|  | 14 | 16 |
|  | 15 | 4 |
|  | 16 | 5 |
|  | 20 | 13 |
|  | Total | 60 |
|  | 1 | 1 |
|  | 2 | 4 |
|  | 3 | 15 |
|  | 5 | 17 |
| Splice donor variants | 8 | 6 |
|  | 13 | 2 |
|  | 14 | 6 |
|  | 15 | 3 |
|  | 16 | 2 |
|  | 20 | 4 |
|  | Total | 39 |
|  | 1 | 3 |
|  | 2 | 2 |
|  | 3 | 8 |
|  | 5 | 7 |
| Splice acceptor variants | 8 | 5 |
|  | 13 | 1 |
|  | 14 | 3 |
|  | 15 | 3 |
|  | 16 | 3 |
|  | 20 | 4 |
|  | Total | 22 |
|  | 1 | 1 |
|  | 2 | 0 |
|  | 3 | 12 |
|  | 5 | 5 |
| Mature miRNA variants | 8 | 1 |
|  | 13 | 0 |
|  | 14 | 2 |
|  | 15 | 1 |
|  | 16 | 0 |
|  | 20 | 0 |
|  | Total | 33 |
|  | 1 | 2 |
|  | 2 | 2 |
|  | 3 | 5 |
|  | 5 | 5 |
| Other variants | 8 | 2 |
|  | 13 | 0 |
|  | 14 | 8 |
|  | 15 | 1 |
|  | 16 | 0 |
|  | 20 | 8 |
